# Outcomes of deferred revascularisation following negative fractional flow reserve in diabetic and non-diabetic patients: a meta-analysis

**DOI:** 10.1186/s12933-023-01751-5

**Published:** 2023-01-30

**Authors:** Avedis Ekmejian, Hari Sritharan, Dinesh Selvakumar, Venkateshka Venkateshka, Usaid Allahwala, Michael Ward, Ravinay Bhindi

**Affiliations:** 1 Department of Cardiology, Interventional Cardiologist, Royal North Shore Hospital, Reserve Rd, St Leonards, Sydney, 2065 Australia; 2 University of Sydney, Camperdown, Australia; 3 Northern Sydney Local Health District Executive, Hornsby, Australia

**Keywords:** FFR, Fractional Flow Reserve, Diabetes, Outcomes, MACE, Negative, Deferred revascularisation

## Abstract

**Background:**

Fractional Flow Reserve (FFR) is a widely applied invasive physiological assessment, endorsed by major guidelines to aid in the decision to perform or defer revascularisation. While a threshold of  > 0.8 has been applied universally, clinical outcomes may be affected by numerous factors, including the presence of diabetes. This meta-analysis aims to investigate the outcomes of diabetic versus non-diabetic patients in whom revascularisation was deferred based on negative FFR.

**Methods:**

We performed a meta-analysis investigating the outcomes of diabetic and non-diabetic patients in whom revascularisation was deferred based on negative FFR. A search was performed on MEDLINE, PubMed and EMBASE, and peer-reviewed studies that reported MACE for diabetic and non-diabetic patients with deferred revascularisation based on FFR  > 0.8 were included. The primary end point was MACE.

**Results:**

The meta-analysis included 7 studies in which 4275 patients had revascularisation deferred based on FFR > 0.8 (1250 diabetic). Follow up occurred over a mean of 3.2 years. Diabetes was associated with a higher odds of MACE (OR = 1.66, 95% CI 1.35–2.04, p =  < 0.001), unplanned revascularisation (OR = 1.48, 95% CI 1.06–2.06, p = 0.02), all-cause mortality (OR = 1.74, 95% CI 1.20–2.52, p = 0.004) and cardiovascular mortality (OR = 2.08, 95% CI 1.07–4.05, p = 0.03).

**Conclusions:**

For patients with stable coronary syndromes and deferred revascularisation based on FFR > 0.8, the presence of diabetes portends an increased long-term risk of MACE compared to non-diabetic patients.

*Trail registration* URL: https://www.crd.york.ac.uk/PROSPERO/; Unique identifier: CRD42022367312.

**Supplementary Information:**

The online version contains supplementary material available at 10.1186/s12933-023-01751-5.

## Introduction

Invasive coronary physiology using hyperaemic and non-hyperaemic indices are guideline-endorsed tool, aiding the decision to revascularise or defer revascularisation in the setting of stable coronary syndromes [1, 2]. Fractional Flow Reserve (FFR) was the first of these modalities to be applied in the clinical setting, with a ratio of  > 0.8 accepted as the threshold for with reduced incidence of major adverse cardiovascular events (MACE) with deferral of revascularisation [3, 4]. For non-hyperaemic indices such as instantaneous wave-free ratio (iFR), the value associated with safe deferral of revascularisation is > 0.89 [5]. FFR_CT_ has an emerging role in the management and prognostication of stable coronary disease [6, 7], and has recently become a guideline-endorsed modality in the evaluation of chest pain [8].

With greater uptake of physiology guided revascularisation, it is now apparent that outcomes following deferred revascularisation may not be equal amongst all patient sub-groups. This is particularly true of diabetic patients, in whom coronary physiology may be confounded by micro-vascular disease or high-risk plaque characteristics [9, 10]. There are multiple trials [11–17] which suggest a higher incidence of MACE in diabetic patients in whom revascularisation was deferred based on negative FFR, however these trials were not powered sufficiently to assess individual endpoints. Indeed, the proportion of diabetic patients in studies specifically investigating the safety of deferral is low, varying between 11.3% in DEFER [18], and 30.4% in DEFINE-FLAIR [5]. Therefore, there remains a role in analysing the pool of data to define the risk of deferring revascularisation for diabetic patients.

In this meta-analysis, the outcomes of diabetic and non-diabetic patients in whom revascularisation was deferred based on negative FFR is investigated, with emphasis on the incidence of long-term MACE.

## Methods

We performed this meta-analysis according to the Preferred Reporting Items for Systematic Reviews and Meta-Analyses (PRISMA) recommendations [19] and the Meta-analysis of Observational Studies in Epidemiology (MOOSE) reporting guidelines [20]. The study protocol was registered in the PROSPERO database (CRD42022367312). We searched for prospective and retrospective studies investigating the outcomes of diabetic or non-diabetic patients in whom revascularisation was deferred based on negative FFR.

### Selection criteria and search strategy

The study designs eligible for inclusion were randomised controlled trials, prospective observational trials, case-control studies, cohort studies and cross-sectional studies. Peer-reviewed studies that reported patient-oriented rather than vessel-oriented MACE for diabetic and non-diabetic patients with deferred revascularisation based on FFR were included. Consistent with current guidelines [1, 2], only the patients with deferred revascularisation using an FFR threshold of > 0.8 were analysed as part of this meta-analysis. For studies in which the pre-defined threshold for deferral of revascularisation was FFR > 0.75 or resting indices (such as iFR), only data for patients with FFR > 0.8 were extracted if possible, otherwise the study was excluded. Publications were also excluded if outcomes were reported with less than one year follow-up, and if hazard ratios were reported rather than raw data. Publications not in English and those with poorly defined follow up periods were also excluded.

Three of the researchers in the study were be allocated to data extraction (A.E, D.S, H.S). Keywords using Medical Subject Heading (MeSH) terms included: FFR; Fractional Flow Reserve; Diabetes; Outcomes; MACE; Negative; Deferred. The search was performed on electronic databases MEDLINE, PubMed and Embase until November 8, 2022. The reviewers documented the number of articles screened and reviewed the abstracts for inclusion and exclusion criteria. Peer-reviewed studies which meet the inclusion and exclusion criteria and which report on the primary and secondary outcomes were included in this meta-analysis.

### Outcomes

The primary outcome was MACE, which comprised a composite of all-cause death, nonfatal spontaneous myocardial infarction (MI, excluding peri-procedural MI) and coronary revascularisation. Secondary outcomes included MI, unplanned revascularisation, all-cause death, and cardiovascular death. Myocardial infarction was defined according to the fourth universal definition [21]. Unplanned revascularisation was defined as revascularisation for a lesion not meeting the ischaemic threshold at the index procedure and not planned for staged revascularisation following the index procedure.

### Risk of bias and quality assessment

Risk of bias and quality assessment was performed for each trial by two independent investigators (A.E and D.S) using the Cochrane Collaboration Framework Risk Of Bias In Non-randomized Studies—of Exposure (ROBINS-E) tool (Additional file 1: Fig. S1) [22]. Discrepancies were resolved by a third independent reviewer (U.A). Publication bias and small-study effects were evaluated using Begg’s test and Funnel Plots for primary and secondary outcomes.

### Data synthesis and statistical analysis

Crude event numbers were analysed because this was reported at defined time intervals. The outcome variables of interest are binary, and therefore, the odds ratio (OR) with the respective 95% confidence interval (CI) was used as a measure of the effect size in the Meta-analysis.

Tests of heterogeneity were conducted using Q statistic, which is distributed as a chi-square random variable (assumption of homogeneity of effect sizes). The between-study heterogeneity was quantified using the I [2] statistic [23], with I^2^ < 50% considered low heterogeneity.

The pooled effect analysis in the Meta-analysis model was performed using a random-effects model, to account for between-study variation and within-study variation [24]. The longitudinal effect of time was assessed using generalised linear mixed effect model based on logit link function within the meta-analysis. Various correlation structures across time assessed in the model specification included—(i) independent, (ii) compound symmetry and (iii) Heteroscedastic auto-regressive of order 1, HAR (1). The best (parsimonious) model performance was assessed using the information theory-based Akai’s Information Criterion (AIC) measure. The results of the included studies are presented in a forest plot. P-values were two-tailed at a 0.05 level of significance (p-values less than 0.05 are considered statistically significant). Meta-analyses were performed in STATA, V17.0 software.

## Results

### Study selection

Our initial search yielded 13423 records, with 2634 duplicates removed, and 2195 removed due to publication date before 2012 (Additional file 1: Fig. S2). 8594 records were screened by title, after which 99 abstracts were reviewed. 24 full texts were assessed for eligibility. Of these, records were excluded if crude event numbers were not reported (n = 9), the FFR threshold for deferral was > 0.75 (n = 2), time to event not reported (n = 4), or the study population was exclusively patients with acute coronary syndromes (n = 2). Following this, 7 studies were found to meet the pre-selection criteria and were included in the meta-analysis. A total of 4275 patients (1250 diabetic and 3025 non-diabetic) were included in this meta-analysis with a mean follow up of 3.02 years.

### Publication Bias

Begg’s test results showed no significant small study effect or publication bias for any outcome (p > 0.05). There was no visually observed publication bias in the Funnel plots for the primary or secondary outcomes (Additional file 1: Figs. S3–S7).

### Baseline Characteristics

Overall, 4275 patients were included in the analysis, including 1250 diabetic patients (29.2%). Most patients had stable disease or non-culprit lesions assessed with FFR. Characteristics of the included studies and patients are summarised in Table 1.

**Table 1:** Characteristics of included studies in meta-analysis

	Van Belle et al [11]	Lee et al [12]	Liu et al [13]	Alkhalil et al [14]	Castro-Meija et al [15]	Banerjee et al [16]	Hoshino et al [17]
Publication Year	2020	2019	2016	2020	2022	2021	2020
Follow up duration (years)	1	1	3	4	4	5	5
Trial Design	Prospective cross-sectional study	Post-hoc analysis of RCT	Prospective Registry	Prospective cross-sectional study	Retrospective open-label	Prospective Cohort Study (sub-group analysis)	Pooled analysis of 3 prospective registries (sub-group analysis)
Pressure wire modality and threshold for deferred revascularisation *	FFR > 0.8	FFR > 0.8; iFR > 0.89	FFR > 0.8	FFR > 0.8	FFR > 0.8; iFR > 0.89	FFR > 0.75	FFR > 0.75
Number of patients	958	579	512	860	434	53	879
Primary Outcome	MACE	MACE	MACE	MACE**	MACE	MACE	MACE
% Diabetic	30.9%	30.6%	27%	18.5%	35.3%	52.8%	33.6%
Indication for physiology	Stable disease (75.3%); Current or recent ACS (24.7%)	Stable disease (81.2%)	Stable disease (60%)	N/A	Stable disease (35.6%); non-culprit vessel in ACS (33.3%); Unstable angina (20%)	Stable disease (100%)	Stable disease or ACS non-culprit vessel
% Male	72.3%	N/A	57%	74%	76.5%	N/A	N/A
Mean Age	66.3	N/A	66.9	66	N/A	N/A	N/A
Mean FFR	0.89 ± 0.05	N/A	N/A	0.88 (0.84–0.91)	0.87 ± 0.46	N/A	N/A

^*^Only patients with FFR > 0.8 used in this meta-analysis

^**^For this study, secondary outcome used in meta-analysis rather than primary outcome, due to consistency with the primary outcome of the meta-analysis

### Outcomes

#### Primary Endpoint

**Fig. 1:**
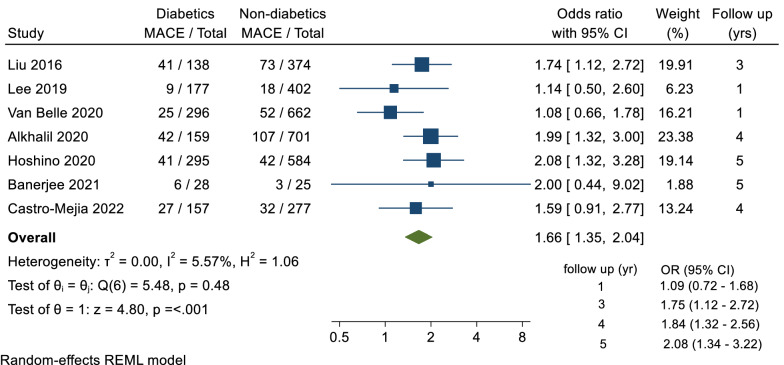
Forest plot of MACE comparison between diabetic and non-diabetic groups

A total of 7 trials reported data for the primary outcome of MACE, including a total of 4275 patients, with a follow-up duration ranging between 1 and 5 years. There was a statistically significant difference in the odds of MACE between diabetics and non-diabetics groups (OR = 1.66, 95% CI 1.35–2.04, p =  < 0.001; I^2^ = 5.57%) (Fig. 1). Longitudinally, there was a significant difference at years 3, 4 and 5 (OR = 1.75, 95% CI 1.12–2.72, OR = 1.84, 95% CI 1.32–2.56 and OR = 2.08, 95% CI 1.34–3.22 respectively) (Additional file 1: Table S1).

#### Myocardial Infarction

**Fig. 2:**
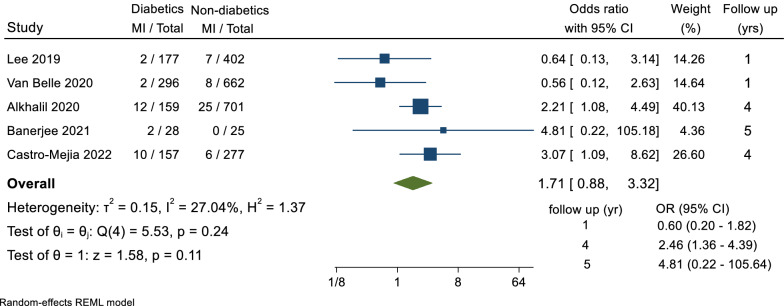
Forest plot of MI event comparison between diabetic and non-diabetic groups

A total of 5 trials reported data for the secondary outcome of MI, including a total of 2884 patients, with a follow-up duration ranging between 1 and 5 years. Overall, there was no significant difference in the odds of MI between diabetics and non-diabetics groups (OR = 1.71, 95% CI 0.88–3.32, p = 0.11, I^2^ = 27.04%) (Fig. 2).

#### Unplanned revascularisation

**Fig. 3:**
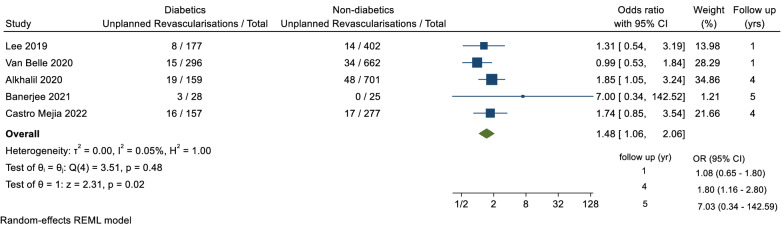
Forest plot of unplanned revascularisation between diabetic and non-diabetic groups

A total of 5 trials reported data for the secondary outcome of urgent revascularisation, including a total of 2884 patients, with a follow-up duration ranging between 1 and 5 years. There was a statistically significant difference in the odds of unplanned revascularisation between diabetics and non-diabetics groups (OR = 1.48, 95% CI 1.06–2.06, p = 0.02, I^2^ = 0.05%) (Fig. 3).

#### All-cause mortality

**Fig. 4:**
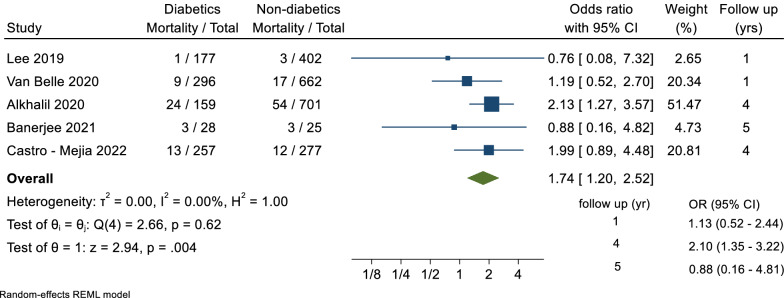
Forest plot of all-cause mortality comparison between diabetic and non-diabetic groups

A total of 5 trials reported data for the secondary outcome of all-cause mortality, including a total of 2884 patients, with a follow-up duration ranging between 1 and 5 years. There was a statistically significant difference in the odds of all-cause mortality between diabetics and non-diabetics groups (OR = 1.74, 95% CI 1.20–2.52, p = 0.004; I^2^ = 0%) (Fig. 4).

#### Cardiovascular mortality

**Fig. 5:**
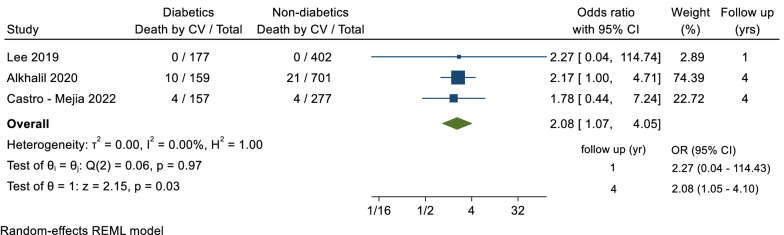
Forest plot of cardiovascular mortality between diabetic and non-diabetic groups

A total of 3 trials reported data for the secondary outcome of cardiovascular mortality, including a total of 1873 patients, with a follow-up duration ranging between 1 and 4 years. Overall, there was a statistically significant difference in the odds of cardiovascular mortality between diabetics and non-diabetics groups (OR = 2.08, 95% CI 1.07–4.05, p = 0.03; I^2^ = 0%) (Fig. 5).

## Discussion

In this meta-analysis involving 4275 patients, the medium to long term incidence of MACE is higher in diabetic patients with deferred revascularisation based on FFR > 0.8 compared to non-diabetic patients, driven by all-cause mortality and unplanned revascularisation. There are various mechanisms which may explain the inferior outcomes in diabetic patients.

### Microvascular Dysfunction

One hypothesis is that FFR may be less reliable in detecting ischaemia in diabetic patients due to the presence of micro-vascular dysfunction [25]. A study by Leung et al [9] has demonstrated higher index of micro-vascular resistance (IMR) in diabetic patients compared to non-diabetic patients. Coronary microvascular dysfunction (CMD) may result in reduced coronary flow and vasodilatory capacity, increasing the chance of generating falsely negative FFR values [26]. Another study by Zhang et al [27] investigated micro-vascular function in diabetic patients with chronic coronary syndromes, using a novel coronary angiography-derived index of microcirculatory resistance (ca-IMR). This study showed a significantly higher incidence of CMD among diabetic patients, and also showed that CMD is an independent predictor of MACE among diabetic patients.

Theoretically, iFR may be a more reliable indicator of ischaemia in the setting of micro-vascular dysfunction. One study in support of this premise is the FIGARO trial [28], in which there was a significantly higher proportion of diabetic patients with iFR positive/FFR negative physiology compared to FFR positive/iFR negative physiology (45% vs 33.2%), with diabetes a statistically significant predictor of iFR positive/FFR negative physiology, which has also been demonstrated in another study [29]. However, in the DEFINE-FLAIR sub-group analysis [12], the risk of MACE was not statistically different between iFR or FFR guidance in the deferred diabetic subgroup (6.8% vs 5.1%; *P* = 0.58). This indicates that microvascular dysfunction alone may not account for the poorer outcomes in diabetics patients.

### Plaque Vulnerability and Progression

Plaque vulnerability is another possible determinant of inferior outcomes in diabetic patients. Though this meta-analysis did not demonstrate a statistically significant difference in MI between diabetic and non-diabetic patients, diabetes was associated with higher incidence of MI at 4 years. An increased incidence of plaque vulnerability or plaque progression in diabetic patients may account for this observation. Multiple studies using optical coherence tomography (OCT) [30] and intravascular ultrasound (IVUS) [31] have demonstrated diabetic patients have a higher incidence of thin-cap fibroatheroma and lipid core, features which are linked to an increased risk of plaque rupture. The COMBINE OCT FFR [32] trial followed patients with diabetes and FFR negative lesions which were also interrogated with OCT. At 18 months, the primary outcome of MACE was significantly higher in patients with thin-cap fibroatheroma (TCFA) compared to TCFA-negative patients (13.1% vs 3.3% respectively; p < 0.001). Although this trial does not compare diabetic to non-diabetic patients, it is plausible that the increased incidence of MI for FFR negative diabetic patients is due to a higher incidence of vulnerable plaque, though a dedicated prospective trial would be needed to confirm this. Research regarding the management of non-obstructive vulnerable plaque has been limited, with a trend towards benefit in one study with Percutaneous Coronary Intervention (PCI) using bio-resorbable vascular scaffolds [33]. By contrast, a study by Zhang et al. showed delayed endothelialisation [34] of drug-eluting stents for patients with TCFA, which may present a risk of stent thrombosis. Medical management using lipid-lowering or anti-inflammatory medications may improve plaque stabilisation and lead to reduced MACE [35]. Therefore, there may be therapeutic targets (either medical or interventional) for patients with negative FFR and TCFA, though more research is needed in this field.

Aggressive plaque progression is also a feature of diabetes. The PARADIGM study [36] showed that diabetes is an independent risk predictor for plaque progression over a median inter-scan period of 3.2 years. A pooled analysis of 5 IVUS trials [31] showed that patients with diabetes had more aggressive progression of percentage and total atheroma volume. This may further account for the increased incidence of revascularisation and long-term MACE for diabetic patients.

### Non-coronary factors

Diabetes has important prognostic implications beyond its impact on the coronary tree. A study by Holland et al [37] showed that diabetic cardiomyopathy, diagnosed using global longitudinal strain (GLS), is associated with a high level of long-term adverse outcomes, including death and hospitalisation. Non-cardiac complications of diabetes may also contribute to the difference in all-cause mortality. Of note, one seminal study has shown that diabetic microvascular complications (DMC) such as retinopathy, nephropathy and neuropathy, are an independent predictor of MACE [38], raising the possibility that patients with DMC have a different substrate of atherosclerotic disease, perhaps relating to increased endothelial impairment, oxidative stress, inflammation and fibrosis. These mechanisms may also be linked to higher prevalence of diastolic dysfunction due to cardiac fibrosis and multivessel CAD.

### Future Directions

This study shows unequal outcomes for diabetic and non-diabetic patients applying the same threshold for deferral of revascularisation. Confirming this hypothesis serves as another step towards improving outcomes for diabetic patients. The benefit of PCI using an FFR-guided strategy has been shown to extend to diabetic patients [4], however our meta-analysis shows that the current thresholds for deferred revascularisation may be inappropriate for diabetic patients. Importantly, PCI for diabetic patients is associated with a higher risk of restenosis [39] which may offset the benefit of revascularisation at a lower threshold for ischaemia, however optimised glycaemic control unequivocally reduces the risk of Target Lesion Revascularisation (TLR) in diabetic patients [40, 41]. Therefore, ongoing research should focus on the impacts on improved glycaemic control on outcomes of deferred revascularisation for diabetic patients. Additionally, a randomised controlled trial would be required to test the hypothesis that outcomes for diabetic patients would improve with revascularisation performed at lower thresholds based on FFR.

### Limitations

There are several limitations of this meta-analysis. Firstly, some data has been extracted from studies with various inclusion criteria, including FFR < 0.75 and iFR < 0.9 as the threshold for ischaemia. Although this meta-analysis only included data for patients with FFR > 0.8 and deferred revascularisation, some studies did not have baseline demographic data for this subset of patients. Therefore, results may confounded by risk factors beyond the presence of diabetes. The observational nature of the included studies means there were unequal distributions of diabetics vs non-diabetics, thereby reducing statistical power and increasing type I error. Another limitation is that very few studies outlined the compliance to optimal medical therapy, and no studies specified the degree of diabetic control. These are important confounding factors, which ideally would be analysed as part of the meta-analysis. Lastly, definitions of revascularisation varied slightly between studies, including “urgent revascularisation,” “any revascularisation” and “target lesion revascularisation.”

## Conclusions

For patients with deferred revascularisation based on FFR > 0.8, the presence of diabetes portends an increased long-term risk of MACE compared to non-diabetic patients, driven by unplanned revascularisation, all-cause mortality, and cardiovascular mortality. This highlights a pitfall of FFR in diabetic patients and serves as impetus to refine the management of diabetic patients with stable coronary disease.

## Supplementary Information

**Additional file 1: Table S1.** Pooled odds ratio effects based on meta-analysis incorporating generalized linear mixed effect model. **Figure S1.** ROBINS-E assessment for included studies. **Figure S2.** The Preferred Reporting Items for Systematic Review and Meta-analysis (PRISMA). **Figure S3.** Funnel plot of MACE between diabetic and non-diabetic groups. **Figure S4.** Funnel plot of meta-analysis of MI between diabetic and non-diabetic groups. **Figure S5.** Funnel plot of unplanned revascularisations between diabetic and non-diabetic groups. **Figure S6.** Funnel plot of all-cause mortality between diabetic and non-diabetic groups. **Figure S7.** Funnel plot of cardiovascular mortality between diabetic and non-diabetic groups.

## Data Availability

All data generated or analysed during this study are included in this published article [and its supplementary information files].

## Abbreviations

FFR: Fractional flow reserve
MACE: Major adverse cardiovascular events
iFR: Instantaneous wave-free ratio
MI: Myocardial infarction
CI: Confidence interval
PRISMA: Preferred reporting items for systematic reviews and meta-analyses
MOOSE: Meta-analysis of observational studies in epidemiology
AIC: Akai’s information criterion
MeSH: Medical subject heading
IMR: Index of micro-vascular resistance
CMD: Coronary microvascular dysfunction
OCT: Optical coherence tomography
IVUS: Intravascular ultrasound
TCFA: Thin-cap fibroatheroma
PCI: Percutaneous coronary intervention
TLR: Target lesion revascularisation
caIMR: Coronary angiography-derived index of micro-vascular resistance
CAD: Coronary artery disease
