# Supplementary material for: Outcomes of deferred revascularisation following negative fractional flow reserve in diabetic and non-diabetic patients: a meta-analysis
Source: Cardiovasc Diabetol. 2023 Jan 30;22:22. doi: 10.1186/s12933-023-01751-5 (PMC9887893; doi:10.1186/s12933-023-01751-5)
Supplement: Supplementary file 1 — Additional file 1: Table S1. Pooled odds ratio effects based on meta-analysis incorporating generalized linear mixed effect model. Figure S1. ROBINS-E assessment for included studies. Figure S2. The Preferred Reporting Items for Systematic Review and Meta-analysis (PRISMA). Figure S3. Funnel plot of MACE between diabetic and non-diabetic groups. Figure S4. Funnel plot of meta-analysis of MI between diabetic and non-diabetic groups. Figure S5. Funnel plot of unplanned revascularisations between diabetic and non-diabetic groups. Figure S6. Funnel plot of all-cause mortality between diabetic and non-diabetic groups. Figure S7. Funnel plot of cardiovascular mortality between diabetic and non-diabetic groups. [file 12933_2023_1751_MOESM1_ESM.docx]

Table S1: Pooled odds ratio effects based on meta-analysis incorporating generalized linear mixed effect model

| Outcome | 1 year follow-up | | 3 years follow-up | | 4 years follow-up | | 5 years follow-up | | Heterogeneity statistics |
| --- | --- | --- | --- | --- | --- | --- | --- | --- | --- |
|  | OR (95% CI) | p | OR (95% CI) | p | OR (95% CI) | p | OR (95% CI) | p |  |
| MACE | 1.09 (0.72 – 1.68) | 0.67 | 1.75 (1.12 – 2.72) | .01 | 1.84 (1.32 – 2.56) | <.01 | 2.08 (1.34 – 3.22) | <0.01 | Q = χ^2^ (6) = 5.48, p = .48, I^2^ = 5.57% |
| MI | 0.60 (0.20 - 1.82) | 0.36 | -- | -- | 2.46 (1.36 – 4.39) | <.01 | 4.81 (0.22 – 105.64) | 0.320 | Q = χ^2^ (4) = 4.33, p = .36, I^2^ = 9.57% |
| Unplanned Revascularisation | 1.08 (0.65 – 1.80) | 0.76 | -- | -- | 1.80 (1.16 – 2.80) | <.01 | 7.03 (0.34 – 142.59) | 0.210 | Q = χ^2^ (4) = 3.51, p = .48, I^2^ = 0.05% |
| All-cause Mortality | 1.13 (0.52 – 2.44) | 0.76 | -- | -- | 2.10 (1.35 – 3.22) | <.01 | 0.88 (0.16 – 4.81) | 0.880 | Q = χ^2^ (4) = 2.66, p = .66, I^2^ = 0.00% |
| Cardiovascular Mortality | 2.27 (0.04 – 114.43) | 0.68 | -- | -- | 2.08 (1.05 – 4.10) | 0.03 | -- | -- | Q = χ^2^ (2) = 0.06, p = .97, I^2^ = 0.00% |

*Note: OR = odds ratio, CI: confidence interval, Q (.) = Cochran’s Q statistic for test of between study heterogeneity.*

Figure S1: ROBINS-E assessment for included studies.


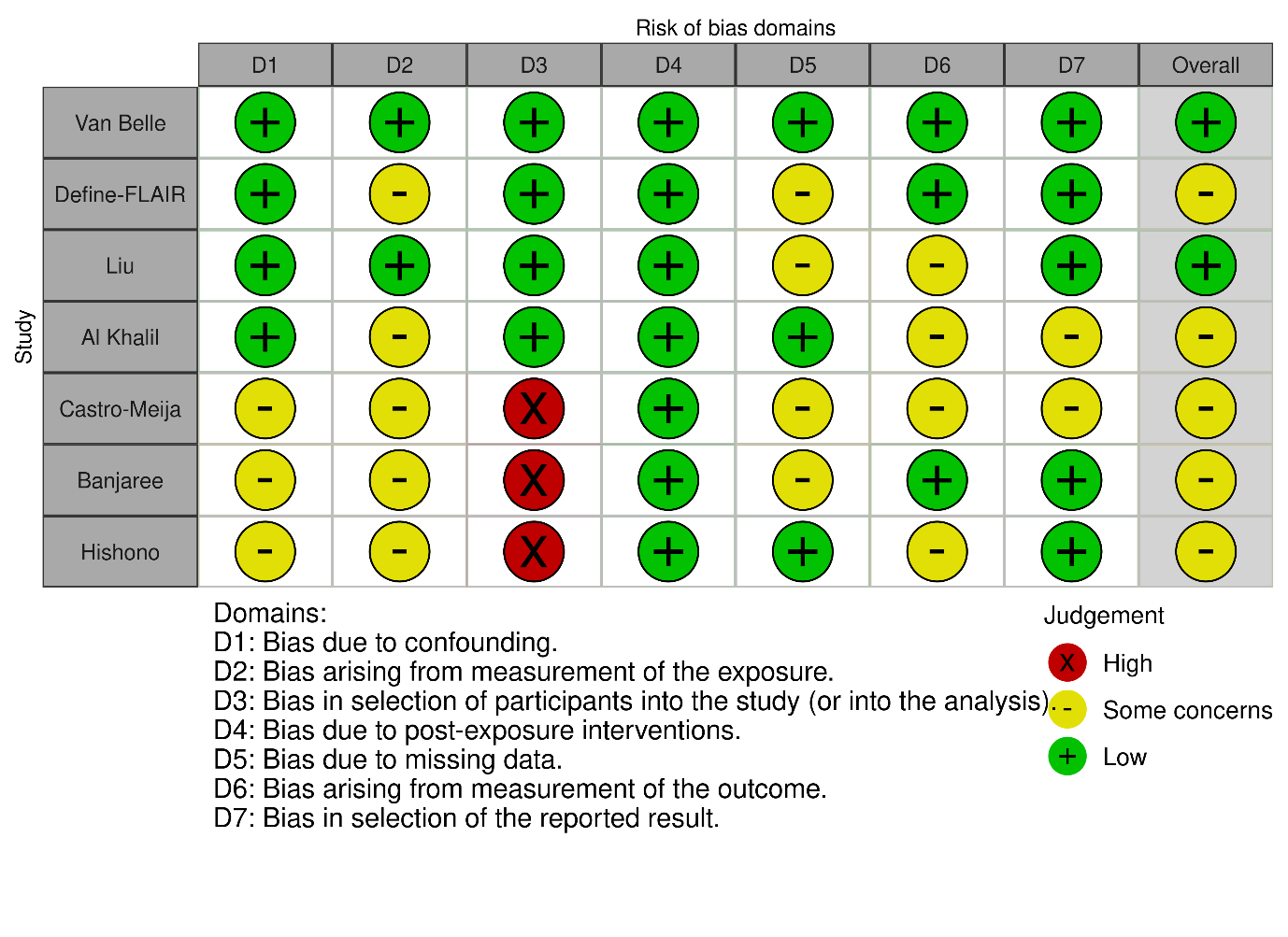


Figure S2: The Preferred Reporting Items for Systematic Review and Meta-analysis (PRISMA).

**Identification of studies via databases and registers**

Records removed *before screening*:

Duplicate records removed (n = 2634)

Records removed due to publication date before 2012 (n =2195)

Records identified from:

Databases (n = 13408)

Additional records through screening reference lists (n = 15)

**Identification**

Records screened

(n = 8594)

Records excluded based on title

(n = 8495)

Reports not retrieved

(n = 0)

Abstracts excluded on screening (n=75)

Reports sought for retrieval

(n = 99)

**Screening**

Reports excluded:

Event numbers not reported (n = 9)

FFR threshold of 0.75 (n = 2)

Time to events not reported, only mean follow up (n=4)

Included patients exclusively with acute coronary syndromes (n=2)

Full manuscripts assessed for eligibility

(n = 24)

Studies included in review

(n = 7)

Reports of included studies

(n = 7)

**Included**

Figure S3: Funnel plot of MACE between diabetic and non-diabetic groups.

Figure S4: Funnel plot of meta-analysis of MI between diabetic and non-diabetic groups.

Figure S5: Funnel plot of unplanned revascularisations between diabetic and non-diabetic groups.

Figure S6: Funnel plot of all-cause mortality between diabetic and non-diabetic groups.

Figure S7: Funnel plot of cardiovascular mortality between diabetic and non-diabetic groups.
